# Naturally Occurring Cholinesterase Inhibitors from Plants, Fungi, Algae, and Animals: A Review of the Most Effective Inhibitors Reported in 2012-2022

**DOI:** 10.2174/1570159X21666230623105929

**Published:** 2023-07-24

**Authors:** Ana Paula Murray, Brunella Biscussi, Valeria Cavallaro, Martina Donozo, Silvana A. Rodriguez

**Affiliations:** 1 INQUISUR-CONICET, Departamento de Química, Universidad Nacional del Sur, Bahía Blanca, Argentina

**Keywords:** Acetylcholinesterase, butyrylcholinesterase, cholinesterase inhibitors, secondary metabolites, Alzheimer’s disease, bioactive compounds

## Abstract

Since the development of the “cholinergic hypothesis” as an important therapeutic approach in the treatment of Alzheimer’s disease (AD), the scientific community has made a remarkable effort to discover new and effective molecules with the ability to inhibit the enzyme acetylcholinesterase (AChE). The natural function of this enzyme is to catalyze the hydrolysis of the neurotransmitter acetylcholine in the brain. Thus, its inhibition increases the levels of this neurochemical and improves the cholinergic functions in patients with AD alleviating the symptoms of this neurological disorder. In recent years, attention has also been focused on the role of another enzyme, butyrylcholinesterase (BChE), mainly in the advanced stages of AD, transforming this enzyme into another target of interest in the search for new anticholinesterase agents. Over the past decades, Nature has proven to be a rich source of bioactive compounds relevant to the discovery of new molecules with potential applications in AD therapy. Bioprospecting of new cholinesterase inhibitors among natural products has led to the discovery of an important number of new AChE and BChE inhibitors that became potential lead compounds for the development of anti-AD drugs. This review summarizes a total of 260 active compounds from 142 studies which correspond to the most relevant (IC_50_ ≤ 15 µM) research work published during 2012-2022 on plant-derived anticholinesterase compounds, as well as several potent inhibitors obtained from other sources like fungi, algae, and animals.

## 1 INTRODUCTION

Alzheimer’s disease (AD) is a progressive neurodegenerative disorder that affects memory, thinking, rationale and language skills that eventually induce personality changes that make the patient unable to take care of themselves. According to the World Health Organization, between 60% and 70% of dementia cases worldwide correspond to AD (https://www.who.int/es/news-room/fact-sheets/detail/dementia). The prevalence of this disease increases dramatically with age, from 3% in people between 65 and 74 years old to 17% for those between 75 and 84 years old and 32% for people over 85 years old [1, 2].

The improvements in healthcare services, the increase in life expectancy and median age have led specialists to estimate that the global number of people living with AD will reach 152 million in 2050. This disease represents not only a financial and social problem to each patient and their family but also for the whole society.

The pathological expression of AD includes extracellular deposits of the amyloid β (Aβ) peptide known as senile plaques or amyloid plaques, intracellular deposits of hyperphosphorylated tau protein (neurofibrillary tangles), neuronal loses and synaptic loses. Over the last years, several research works have described different underlying pathogenic mechanism for AD that comprises neuroinflammation, oxidative stress, the calcium theory, dysregulation of iron metabolism, reduced glucose utilization, imperfect insulin signaling, abnormal cholesterol homeostasis, and mitochondrial dysfunction [2-4]. The most important neurochemical deficiency in AD is the decreased concentration of the neurotransmitter acetylcholine (ACh). The “cholinergic hypothesis” is based on the fact that ACh deficiency is critical in the onset of AD symptoms. This neurotransmitter is used by cholinergic neurons in the peripheral and central nervous systems to mediate synaptic activity. It binds to nicotinic and muscarinic receptors on the postsynaptic membrane transmitting the nerve signal. The enzyme acetylcholinesterase (AChE, E.C. 3.1.1.7), also located in the postsynaptic membrane, catalyzes the breakdown of ACh, and choline molecules are reabsorbed by the presynaptic neuron [5, 6]. AChE has proved to be the most important therapeutic target for symptomatic improvement in AD, since its inhibition is a feasible therapeutic target. Most AChE inhibitors interact with the catalytic site of the enzyme (CAS) located at the bottom of the gorge, where the hydrolysis of ACh takes place [7]. The entrance of this gorge contains the peripheral anionic site (PAS), which can also be targeted, either separately or simultaneously, by potential inhibitors [8]. Furthermore, several studies have shown that AChE interacts with the Aβ peptide *via* hydrophobic amino acids located in the PAS of the enzyme, inducing the formation of Aβ fibrils, thus contributing to the accumulation of senile plaques. These studies indicate that AChE may also serve noncholinergic functions in AD [9]. The inhibition of AChE by blocking the PAS could affect the deposition of this toxic peptide induced by the enzyme, leading to a reduction in the expression of one of the pathologies of the disease. Based on these premises, new classes of PAS-targeting AChE inhibitors have emerged as promising disease-modifying anti-Alzheimer drug candidates [10].

There is another enzyme, butyrylcholinesterase (BChE E.C. 3.1.1.8), known as ‘pseudocholinesterase’, which is also capable of hydrolyzing ACh. While AChE is mainly associated with neurons and axons, BChE is expressed and secreted by glial cells in the brain. In a healthy brain, AChE and BChE are found in a 4:1 ratio [11]. However, in the brains of patients with AD, AChE activity can decrease by up to 45% during the course of the disease, while BChE activity can even double [12].

These two enzymes share about 54% amino acid sequence identity but differ in their specificity towards various substrates and inhibitors [13]. Both enzymes, AChE and BChE, interact with Aβ peptide fibrils. However, BChE does not produce an observable effect on the formation of senile plaques [14]. In BChE the PAS, which mediates substrate activation, has a weaker affinity than PAS in AChE due to the different amino acids present in that region of each enzyme [15].

Taking this background into account, it has been established that a selective, reversible inhibition of BChE or dual reversible inhibitors of AChE and BChE could be crucial in the pathogenesis of middle to advanced stages of AD, in order to prevent a further decline in the cognitive and mental abilities while the decline of cholinergic neurons persists [14, 16, 17].

**Fig. (1):**
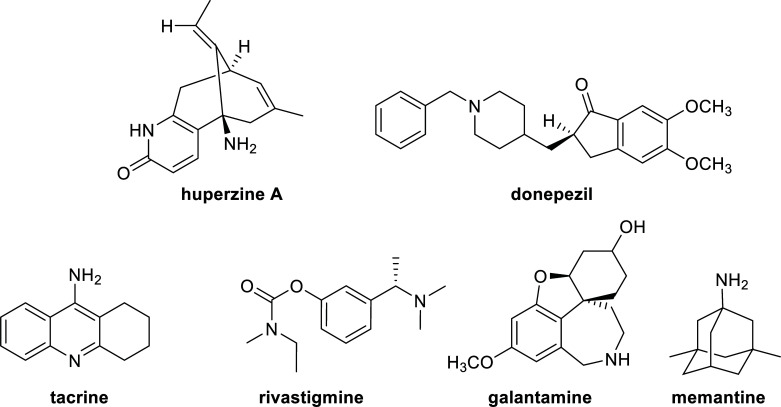
Drugs used for the treatment of AD.

There are currently five drugs approved by international regulatory agencies such as the US-FDA (United States Food and Drug Administration) and the EMA (European Medicines Agency) for the treatment of the cognitive manifestations of AD and the improvement of the quality of life of patients: tacrine (now withdrawn from the market), donepezil, rivastigmine, and galantamine as reversible AChE inhibitors, and memantine as an N-methyl-D-aspartate receptor antagonist (Fig. **1**) [5, 6, 18].

A few known cholinesterase inhibitors have been derived from natural products. For example, the alkaloid galantamine (Fig. **1**), one of the approved cholinesterase inhibitors, was first isolated from the snowdrop (*Galanthus* spp.) and can also be found in other species of the *Amaryllidaceae* plant family, like Narcissus species (*Narcissus* spp.) and snowflake (*Leucojum* spp.) [19]. Huperzine A (Fig. **1**), an alkaloid found in *Huperzia* spp. (Lycopodiaceae), is an AChE inhibitor (AChEi) commercialized as a dietary supplement for memory support. This alkaloid has also shown some beneficial cognitive effect in patients with mild to moderate AD and neuroprotective effects against β-amyloid induced oxidative injury and a reduction of oxidative stress in a few trials [20].

These effective AChEi have inspired the search for other naturally occurring compounds with potential applications in AD therapy. These studies led to the discovery of an important number of secondary metabolites with the ability to inhibit AChE and/or BChE and a substantial number of research papers have been published in this field during the last decades.

Several reviews on the newly discovered AChEi obtained from plants, fungus and marine organisms have been published over the last decades, including alkaloids, terpenoids, flavonoids and other phenolic compounds [16, 21-24]. Interestingly, although the literature demonstrates to be rich in studies about AChEi obtained from natural sources, this issue keeps on being the center of attention for research, as confirmed by the increasing number of studies published every year. Therefore, the purpose of this review is to continue and update our previous work published in 2013 on this matter [25]. Here, we intend to provide a comprehensive summary of the literature published during 2012-2022 on plant-derived compounds, as well as fungal, algae and animal metabolites, which have been reported to inhibit cholinesterase. For the sake of brevity and to focus our attention on the most relevant findings, only molecules with IC_50_ ≤ 15 µM have been considered (for any of the enzymes). Furthermore, most of the results on cholinesterase inhibition included in the present review refer to *in vitro* assays conducted with AChE from electric eel or BChE from equine serum, unless otherwise stated.

## 2 CHOLINESTERASE INHIBITORS FROM PLANTS

Considering the excellent cholinesterase inhibition elicited by galantamine and huperzine A it is not a surprise that these two families and their phytochemistry have been thoroughly studied in the past [21, 26].

**Fig. (2):**
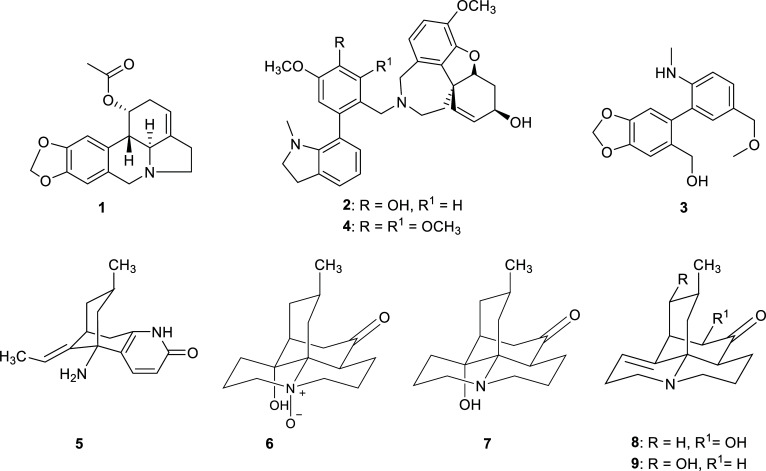
Cholinesterase inhibitors reported for Amaryllidaceae and Lycopodiaceae families.

A survey of the literature shows that in the last decade, only a few records have been found for AChEi obtained from plants belonging to the Amaryllidaceae family (Table **1**). Four compounds (**1**-**4**) with potent cholinesterase inhibition were obtained from four species of this family (Fig. **2**). Among them, the best enzymatic inhibition was observed for narciabduliine (**2**) isolated from *Narcissus pseudonarcissus* [27], and for narcieliine (**4**), found in *Zephyranthes citrina,* both alkaloids with a narcikachnine-type structure. Compound **2** resulted in being a dual inhibitor of both AChE and BChE, while compound **4** showed selective BChE inhibition [28].

**Fig. (3):**
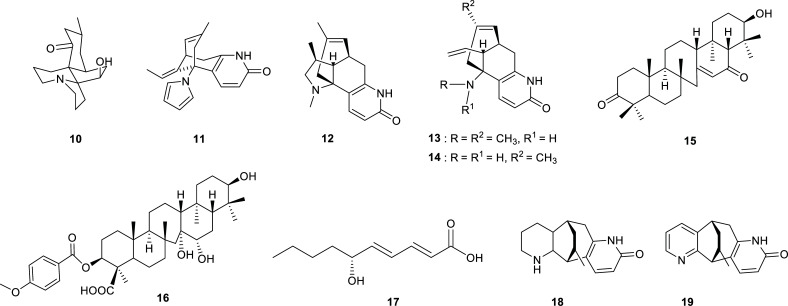
Cholinesterase inhibitors reported for Lycopodiaceae family.

In the Lycopodiaceae family (Table **1**), it is worth mentioning the results reported by Thorroad *et al.* for the species *Huperzia carrinata*, which presents lycodine- and lycopodine-type alkaloid with potent AChE inhibition [29]. The most effective AChEi was 12-epilycodine *N*-oxide (**6**) (Fig. **2**) with a submicromolar IC_50_ value. A strong AChE inhibition was also detected for huperzine C (**14**), another lycodine-type alkaloid obtained from *Lycopodiastrum casuarinoides* (Fig. **3**) [30]. Surprisingly, the best AChE inhibition registered in the Lycopodiaceae family was not observed for an alkaloid but rather for an unsaturated fatty acid (**17**), isolated from *Lycopodiella cernua* (Fig. **3**) [31].

**Fig. (4):**
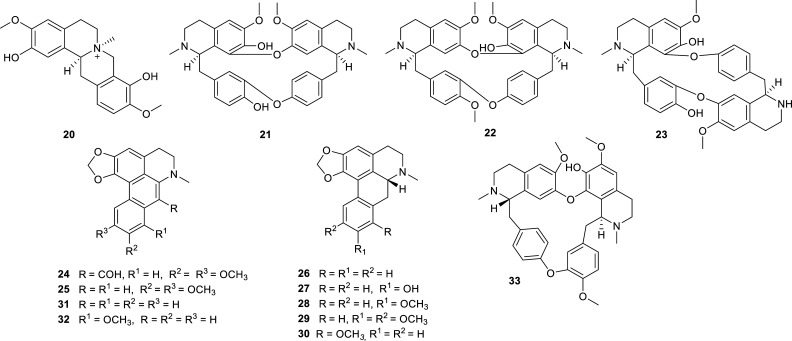
Cholinesterase inhibitors reported for Menispermaceae family.

The Menispermaceae family is one of the families that has provided more cholinesterase inhibitors during the last decade, thanks to the studies of the plants *Cissampelos pareira*, *Stephania epigaea*, *S. pierrei* and *S. tetrandra* (Table **2**). Several aporphine alkaloids and bisbenzylisoquinoline alkaloids with potent AChE, and in some cases also BChE inhibition have been reported (Fig. **4**). Among them, the best AChE inhibition was observed for dehydroroemerine (**31**), with an IC_50_ value of 1.21 ± 0.09 µM, while (-)-stephanine (**30**) resulted in being the more potent inhibitor of BChE (IC_50_ = 2.80 ± 0.07 µM). Both alkaloids, **30** and **31**, were isolated from the tubers of *S. pierrei*, a Thai medicinal plant used to treat body edema, migraine, and heart disease [32].

**Fig. (5):**
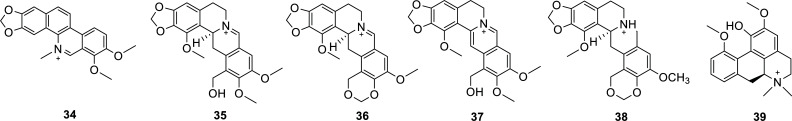
Cholinesterase inhibitors reported for Papaveraceae family.

Plants of the Papaveraceae family are known to produce alkaloids with biological activities related to cholinesterase inhibition, neuroprotection or analgesic activity [33]. However, only two studies are found in the literature in the period covered by this review that are worth to be mentioned (Table **3**). Some bioactive alkaloids were obtained from *Chelidonium majus* and *Papaver setiferum* [34, 35], of which the best activity was observed for chelerythrine (**34**) and 7,8-didehydroorientalidine (**36**) (Fig. **5**). Compound **34** was evaluated with electric eel and human AChE, and equine and human BChE, showing good results in every assay. Also, this compound was identified as a highly active inhibitor of Aβ_1-40_ aggregation and as a molecule able to disaggregate preformed Aβ aggregates [34]. On the other hand, compound **36**, a new alkaloid isolated as a stable trifluoroacetic acid salt from the capsules of the common ornamental poppy *P. setiferum,* selectively inhibited AChE [35].

**Fig. (6):**
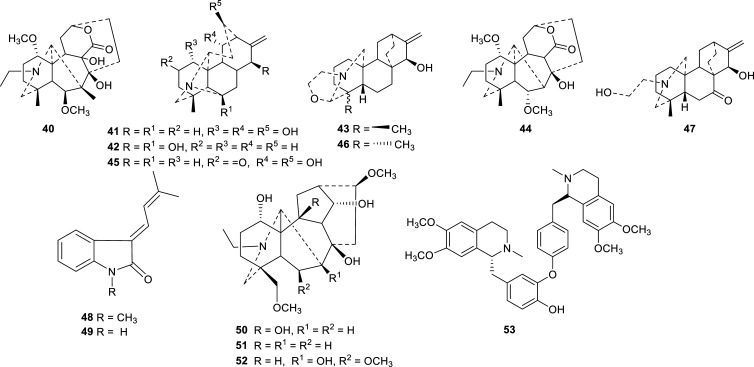
Cholinesterase inhibitors reported for Ranunculaceae family.

Many terpenoidal alkaloids have been reported as bioactive compounds isolated mainly from medicinal plants of the genera *Ranunculus*, *Delphinium*, *Clematis,* and *Aconitum,* of the Ranunculaceae family [36]. During the last decade, several alkaloids with the ability to inhibit cholinesterase were reported to be present in four plants of this family (Table **4**, Fig. **6**). Most of them are terpenoidal alkaloids with moderate activity towards AChE and BChE, except for dauricine (**53**), which selectively inhibit AChE with an IC_50_ value of 1.41 ± 0.02 µM. This bisbenzylisoquinoline alkaloid was found in the roots of *Dichocarpum auriculatum,* a plant that grows in southwestern China, where it is locally used in folk medicine to treat epilepsy [37].

**Fig. (7):**
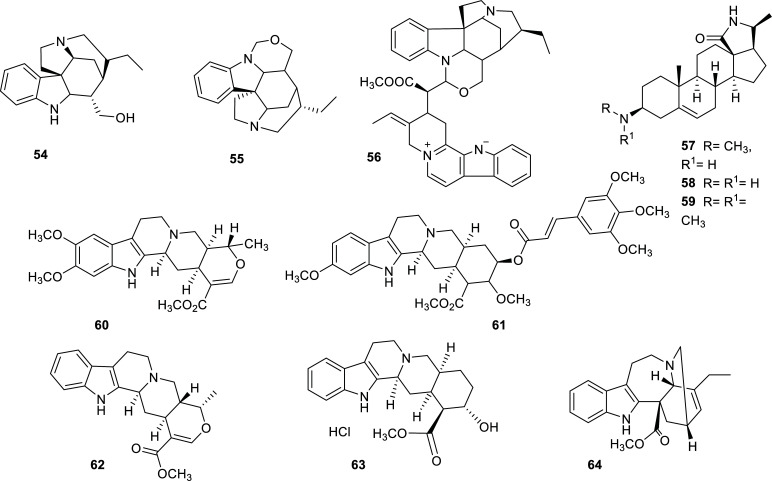
Cholinesterase inhibitors reported for Apocynaceae family.

The Apocynaceae family is a large family of plants, some of them with medicinal properties, which has provided some strong AChE inhibitors in the past. Examples are the alkaloids isolated from *Catharanthus roseus*, *Ervatamia hainanensis*, *Tabernaemontana australis*, *T. divaricate*, and *Holarrhena antidysenterica* [25]. Recently, six species belonging to this family have contributed to the discovery of potent cholinesterase inhibitors (Table **5**). The most effective inhibitors isolated from this family are indole and steroidal alkaloids (Fig. **7**). A powerful inhibitor of both cholinesterases was obtained from an active extract of the stembarks of *Geissospermum vellosii,* a Brazilian tree [38]. This inhibitor was identified as an indole alkaloid named 3′,4′,5′,6′-tetradehy-drogeissospermine (**56**) that presented IC_50_ values of 0.45 ± 0.01 µM and 0.32 ± 0.02 µM for AChE and BChE, respectively. From the same species, the alkaloid geissoschizoline (**54**) was also isolated, which presented a lower but still powerful enzymatic inhibition with the advantage of not being cytotoxic in cellular assays. This indole alkaloid was also active in human cholinesterases and showed promising anti-inflammatory activity [38]. Another potent AChE inhibitor reported in plants of this family was mokluangin C (**58**), a new steroidal alkaloid isolated from *Holarrhena pubescens* that showed an IC_50_ value of 1.44 ± 0.66 µM [39].

**Fig. (8):**
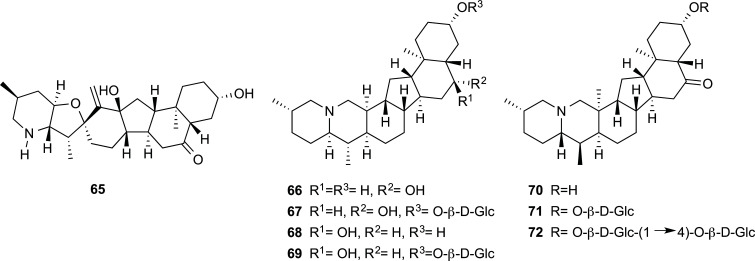
Cholinesterase inhibitors reported for Liliaceae family.

The genus *Fritillaria* (Liliacea) is a known source of isosteroidal alkaloids that have proven to be able to inhibit cholinesterase [22]. In 2012-2022, several active alkaloids of this type, some of them new, were isolated from bulbs of *F. walujewii* (Fig. **8**) [40]. These alkaloids elicited moderated inhibition against AChE and BChE, tortifoline (**66**) being the most effective against both enzymes (Table **5**). This alkaloid had been previously isolated form *F. tortifolia* [41].

**Fig. (9):**
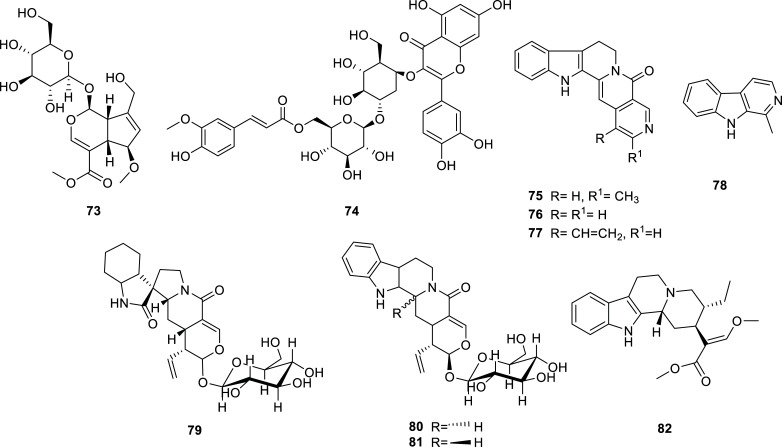
Cholinesterase inhibitors reported for Rubiaceae family.

In the Rubiaceae family, there are some plants that are known for their medicinal properties (*Cinchona officinalis*, *Carapichea ipecacuanha*, and *Psychotria viridis*), their ornamental uses (*Gardenia jasminoides*) or as natural dyes (*Rubia tinctorum* and *Morinda citrifolia*). In the period 2012-2022 some cholinesterase inhibitors were obtained from four species belonging to this family (Fig. **9**). Most of these compounds were monoterpene-indole alkaloids with the ability to selectively inhibit BChE or be more active to this enzyme rather than to AChE (Table **6**). Angustidine (**75**), isolated from the bark of *Nauclea officinalis,* was the most potent BChE inhibitor (IC_50_ = 1.03 µM), while 7-epi-javaniside (**79**) was an efficient inhibitor of both AChE and BChE con IC_50_ values of 2.85 ± 0.50 µM and 2.13 ± 0.10 µM, respectively [42, 43].

**Fig. (10):**
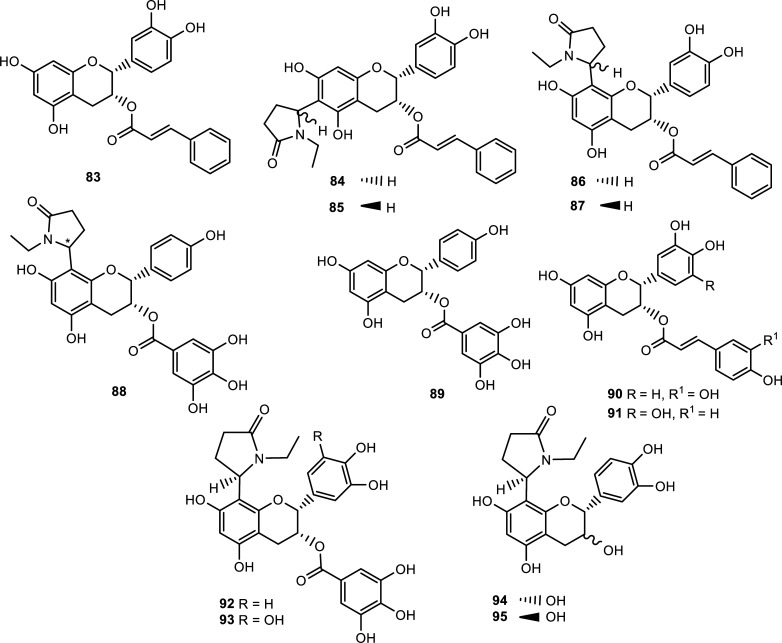
Cholinesterase inhibitors reported for Theaceae family.

Bioprospection in plants that are usually consumed as food or beverage is always of scientific interest. The plant *Camellia sinensis* (Theaceae), whose leaves and leaf buds are used to produce the popular beverage tea, has been thoroughly studied in the search for bioactive compounds [44]. In recent years, several publications have reported that this plant contains flavanols and flavoalkaloids that are potent AChEi (Table **7**, Fig. **10**). The best inhibition results were informed by Gaur and co-workers for some novel cinnamoylated epicathechins (**84**-**87**) that were first prepared and later detected in leaves of tea cultivars [45]. These tea secondary metabolites inhibited AChE with submicromolar IC_50_ values (0.14-0.21 µM).

**Fig. (11):**
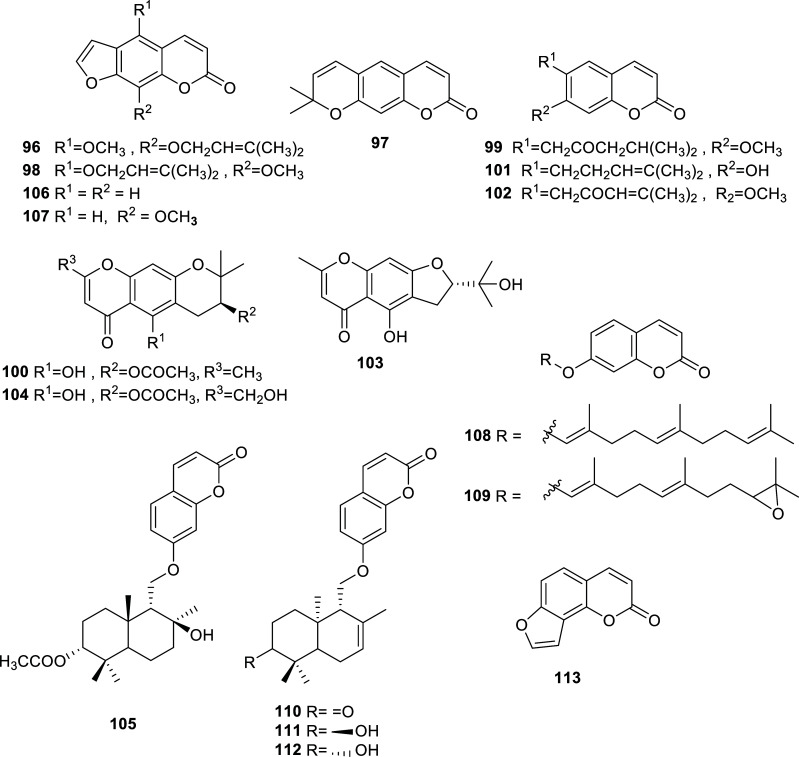
Cholinesterase inhibitors reported for Apiaceae family.

Apiaceae is a large family that comprises more than 3000 species cultivated worldwide, mostly aromatic plants, that are commonly used as food, flavors, ornamental plants, and/or traditional ethnomedicines [46, 47]. Many Apiaceae plants of the genera *Angelica* and *Ferula* are rich in phytochemicals with valuable biological activities such as antioxidant, antimicrobial, anti-inflammatory, antidiabetic, anticarcinogenic, and cardioprotective properties [48]. Recently, some plants of this family have afforded several effective cholinesterase inhibitors, usually coumarins and chromones (Table **8**, Fig. **11**). From these studies, it is to highlight the activity of xanthoxin (**107**) and umbelliprenin (**108**), that resulted in being the best inhibitors of AChE and BChE, with IC_50_ values of 0.76 ± 0.3 and 1.10 ± 0.19 µM, respectively. Compound **107** was obtained from the roots of *F. lutea,* while compound **108** was isolated from the fruits of *Heptaptera cilicica* [49, 50].

**Fig. (12):**
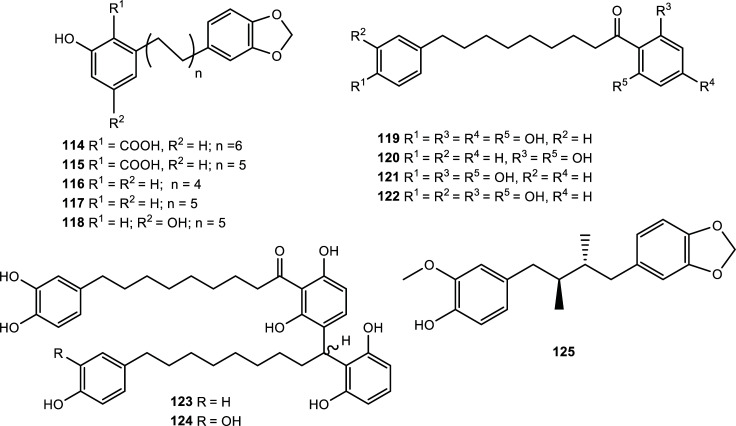
Cholinesterase inhibitors reported for Myristicaceae family.

**Fig. (13):**
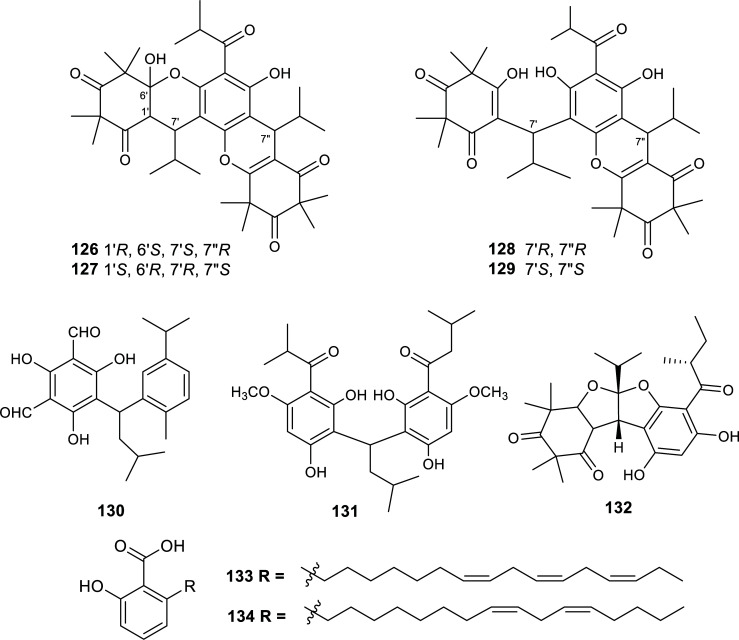
Cholinesterase inhibitors reported for Myrtaceae family.

Various anacardic acid derivatives, cardanol derivatives, acylphenols and acylphloroglucinols showing cholinesterase inhibition have been described as bioactive metabolites isolated from plants belonging to the Myristicaceae and Myrtaceae families (Table **9**, Figs. **12** and **13**). The best results for the Myristicaceae metabolites were registered for the acyl phenols malabaricone A (**120**), malabaricone B (**121**) and malabaricone C (**122**), isolated from the ethyl acetate extract of the fruits of *Myristica cinnamomea,* which presented IC_50_ values between 1.3 and 1.9 µM for AChE. It is to note that while compounds **121** and **122** also inhibited BChE successfully, compound **120** acted as a selective AChE inhibitor [51]. *M. cinnamomea* is closely related to *M. fragrans* Houtt. (nutmeg) whose secondary metabolites are memory enhancers. Also, significant AChE inhibitory activity had been previously informed for acylphenols isolated from *M. crassa* and for the extract of *M. fragrans* [25].

In the case of inhibitors informed for the Myrtaceae family, the most effective was anacardic acid C (**134**), which presented an IC_50_ value of 0.54 µM when it was evaluated with AChE [52]. This phenolic lipid was obtained as one of the active principles of the hexane extract of *Syzygium jambos* (Myrtaceae), a Malaysian plant used in folk medicine for its antipyretic and anti-inflammatory properties.

**Fig. (14):**
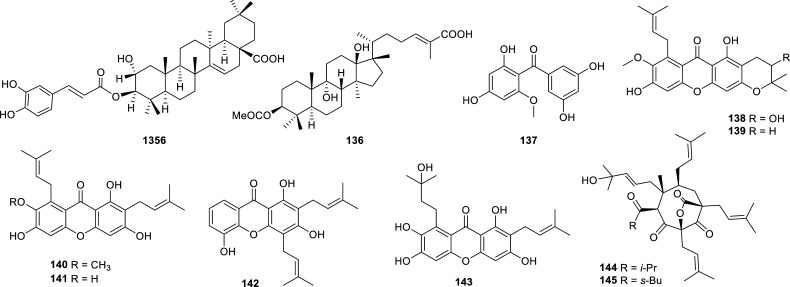
Cholinesterase inhibitors reported for Clusiaceae family.

Plants within the Clusiaseae family are known to produce a wide range of phytochemicals like isoprenylated xanthones, biflavonoids and anthraquinones. Many species of this family exhibit anti-inflammatory or immunosuppressive activities and some species are also known for their use in folk medicine, their horticultural value or their edible fruits [53]. In recent years, three species of this family were reported in the literature due to their activity against cholinesterase (Table **10**, Fig. **14**). In particular, an extract of *Garcinia mangostana* fruit hulls afforded several prenylated xanthones with potent inhibition of both AChE and BChE [54]. While garcinone C (**143**) functioned as a potent AChE inhibitor (IC_50_ = 1.24 µM) and a moderate BChE inhibitor, γ-mangostin (**141**) successfully inhibited both enzymes (IC_50 AChE_ = 1.31 µM, IC_50 BChE_ = 1.78 µM).

**Fig. (15):**
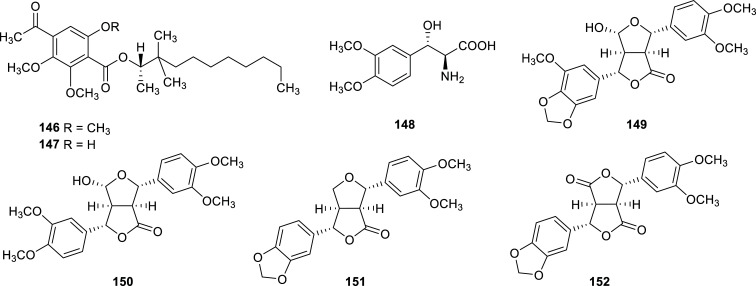
Cholinesterase inhibitors reported for Scrophulariaceae family.

Recently, two plants of the Scrophulariaceae family have been reported to produce cholinesterase inhibitors (Table **11**, Fig. **15**). From these two species, the most interesting is *Leucophyllum ambiguum,* a Mexican plant studied by Rios and co-workers [55]. The authors informed the isolation of four new furofuranoid lignan ciquitins A-D (**149**-**152**) and an α-amino acid (**148**) with very potent AChE inhibition (IC_50_ = 0.001-2.23 µM) from the aerial parts of this plant.

During the period that was examined for this revision (2012-2022), a remarkable number of publications discussed the identification and isolation of natural cholinesterase inhibitors of different compound classes. These secondary metabolites were obtained from a diverse group of plants belonging to different plant families. Those families that provided results for several species, or that afforded many active compounds were analyzed separately (Tables **1-11**). In many cases, just one or two plants of a determined family, giving few (1-3) active compounds, are worth to be mentioned here due to their cholinesterase inhibition potency. For the sake of brevity, these findings have been compiled into two groups, alkaloids (Table **12**) and polyphenols and miscellaneous compounds (Table **13**).

**Fig. (16):**
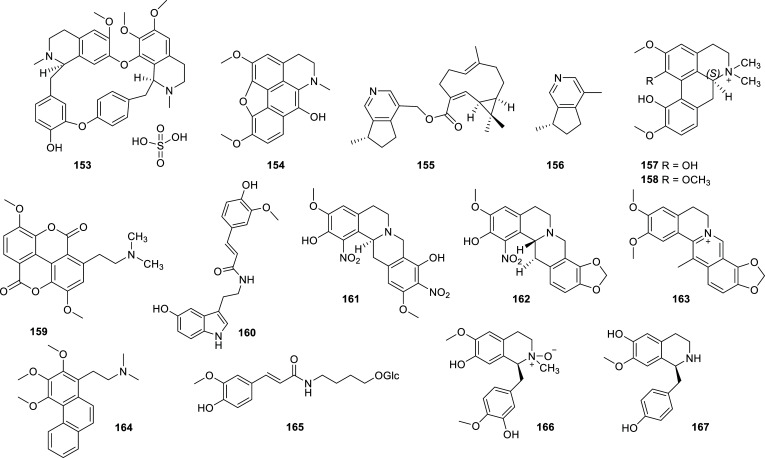
Cholinesterase inhibitors alkaloids obtained from different plant families.

**Fig. (17):**
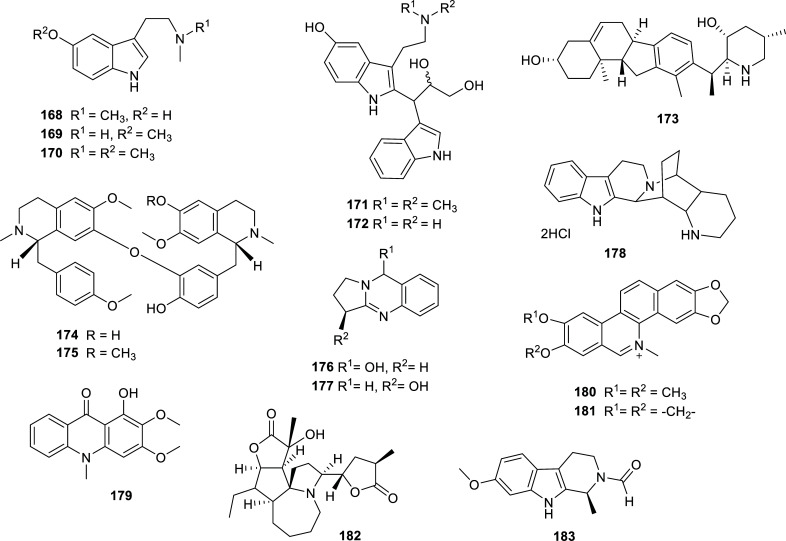
Cholinesterase inhibitors alkaloids obtained from different plant families.

Many alkaloids of different kinds have been isolated from plants, a considerable number of them with good or excellent ability to inhibit AChE and/or BChE. These results are summarized in Table **12** and Figs. **16** and **17**. From these alkaloids, it is to note the potent enzymatic inhibition elicited by liensinine (**175**) and avicine (**181**) (Fig. **17**). Compound **175** is a bisbenzylisoquinoline alkaloid isolated from *Nelumbo nucifera* (Nelumbonaceae) that showed an excellent AChE inhibition (IC_50_ = 0.34 ± 0.02 µM) and potent BChE inhibition [56]. *N. nucifera* is a well-known medicinal plant, commonly known as “sacred lotus” that has been studied due to its therapeutic potential and had already been reported as a source of AChEi [25, 57]. Liensinine (**175**), obtained from active extracts of *N. nucifera* embryos, also exerted significant BACE1 and ONOO¯ scavenging effect, which led the authors of this work to propose that it may act as a multitarget therapeutic or preventive agent for AD [56].

The benzophenanthridine alkaloid avicine (**181**) was also identified as a potent multifunctional candidate isolated from the root extract of *Zanthoxylum rigidum* (Rutaceae). This compound showed dual cholinesterase inhibition, being more active against AChE over BChE, with IC_50_ values in the submicromolar range (IC_50AChE_ = 0.15 ± 0.01 µM, IC_50BChE_ 0.88 ± 0.08 µM) [58]. AChE inhibition was confirmed with human recombinant enzyme (IC_50_*_Hr_*_AChE_ = 0.52 ± 0.05 µM). In addition, this alkaloid presented moderate Aβ1-42 anti-aggregation activity (IC_50_ = 5.56 ± 0.94) and MAO-A inhibition (IC_50_ = 0.41 ± 0.02 μM).

**Fig. (18):**
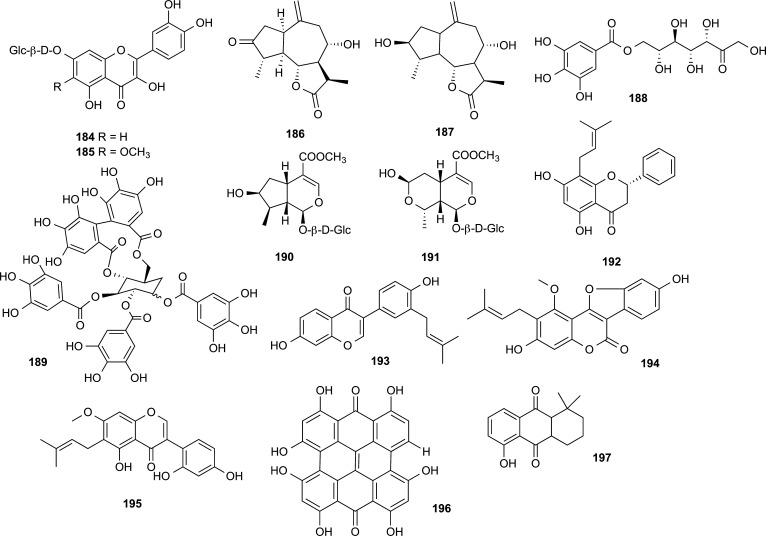
Compounds reported as potent cholinesterase inhibitors isolated from plants of Asteraceae, Cornaceae, Fabaceae, Hypericaceae and Juglandaceae families.

On the other hand, a considerable number of polyphenolic compounds and miscellaneous structural type compounds have also proven to be effective cholinesterase inhibitors. Those that were reported in the literature between 2012 and 2022 are included in Table **13** and displayed in Figs. (**18**, **19** and **20**). From these results, some inhibitors can be highlighted due to their potency (IC_50_ ˂ 1 µM). For instance, two sesquiterpene lactones isolated from *Volutaria abyssinica* (Asteraceae), amberboin (**186**) and lipidiol (**187**) (Fig. **18**) [59]. Both compounds acted like promising AChEi with IC_50_ values of 0.79 ± 0.03 μM and 0.52 ± 0.01 μM for **186** and **187**, respectively. While lactone **186** also effectively inhibited BChE (IC_50_ = 0.58 ± 0.13 μM), compound **187** resulted in being a selective AChEi. Another selective AChEi, an iridoid glycoside named loganin (**190**), was obtained from the fruit of *Cornus officinalis* (Cornaceae) (Fig. **18**). Although it was already known that this compound exhibits immune-regulating, anti-inflammatory, and hepato-protective activity, Bhakta and co-workers found that it also presents powerful AChE inhibition, significant BACE1 inhibition and inhibitory activity against ONOO^-^-mediated tyrosine nitration [60, 61].

Finally, among all the inhibitors summarized in Table **13**, it is to point up the excellent enzymatic inhibition, shown by two of the secondary metabolites afforded by the Fabaceae family, with low nanomolar IC_50_ values. The prenylated flavanone glabranin (**192**), a component of the roots of *Dalea elegans*, has shown a potent AChE inhibition (IC_50_ = 6 ± 1 nM), 12-fold more effective than that reference inhibitor physostigmine, as well as neuroprotective effects against oxidative stress-induced death in both models, granular cerebellar neurons and (NGF)-differentiated PC12 cell [62].

Neobavaisoflavone (**193**) is a natural product found in *Cullen corylifolium* and other plants of the Fabaceae family. This isoflavonoid proved to be even more potent AChEi (IC_50_ = 3 nM) than compound **192**, as well as being able to inhibit BChE very effectively (IC_50_ = 76 nM) [63]. Compound **193** also showed significant cytotoxic activities against common human glioma cancer cell lines and radical scavenging ability (Fig. **18**).

## 3 CHOLINESTERASE INHIBITORS FROM FUNGI, ALGAE, AND ANIMALS

**Fig. (21):**
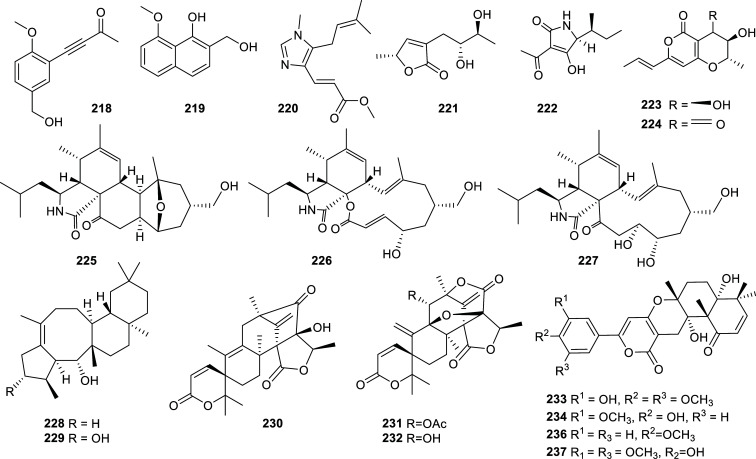
Compounds reported as potent cholinesterase inhibitors isolated from Fungi.

**Fig. (22):**
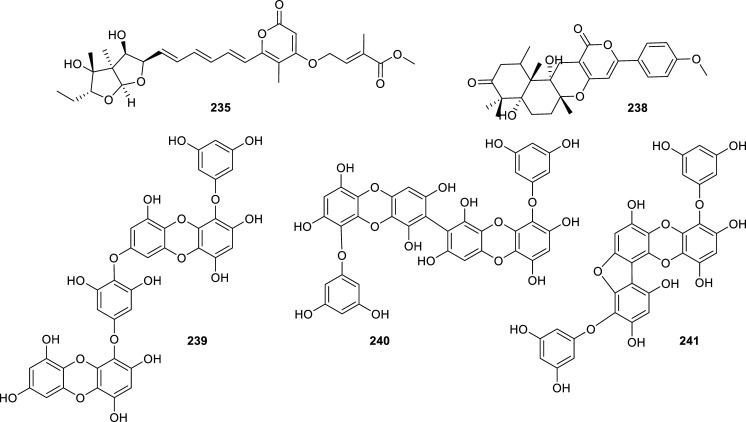
Compounds reported as potent cholinesterase inhibitors isolated from Fungi (cont) and Algae.

**Fig. (23):**
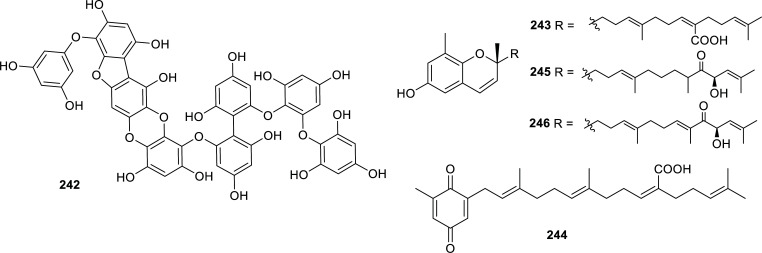
Compounds reported as potent cholinesterase inhibitors isolated from Algae.

**Fig. (24):**
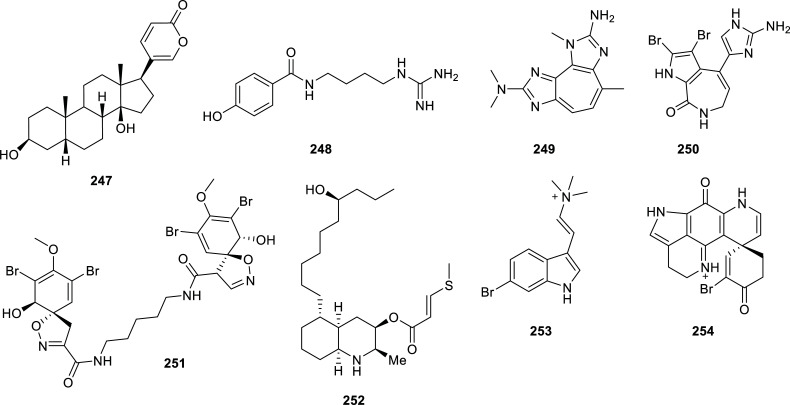
Compounds reported as potent cholinesterase inhibitors isolated from animals.

**Fig. (25):**
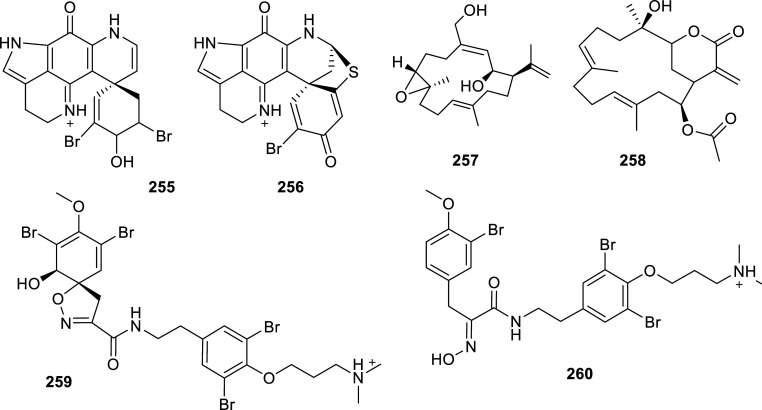
Compounds reported as potent cholinesterase inhibitors isolated from animals.

Even though most of the reported natural cholinesterase inhibitors have been isolated from plants, there are some examples of natural inhibitors obtained from other sources. Those cholinesterase inhibitors found in fungi and algae during the last decade are presented in Table **14** and Figs. **21**, **22** and **23**. Also, several examples of natural inhibitors from animal sources have been discovered over this period and are summarized in Table **15** and Figs. **24** and **25**. These findings are predominantly from marine environments.

The best BChE inhibitor of fungal origin is the alkaloid fungerin (**220**) which selectively inhibited this enzyme over AChE, with an IC_50_ value of 1.75 ± 0.59 µM [64]. This imidazole-type alkaloid was first isolated from a *Fusarium* sp. by Kato *et al.* and identified as an antifungal compound [65]. Other fungal metabolites that showed potent cholinesterase inhibition were the cytochalasan-type alkaloids **225**-**227** produced by the endophytic fungus *Westerdykella nigra* (Sporormiaceae). These compounds inhibited AChE with IC_50_ values in the nanomolar order (Table **14**, Fig. **21**). Several active compounds have been obtained recently from different *Aspergillus* strains but the meroterpenoids territrem D (**233**) and E (**234**) are by far the most potent AChE inhibitors obtained from these fungi, with IC_50_ values of 4.2 and 4.5 nM, respectively [66]. These new lactones were obtained from the marine-derived fungus *Aspergillus terreus* SCSGAF0162 under solid-state fermentation of rice.

Among the secondary metabolites produced by algae, it is to note the activity of phlorotannins like phlorofurofukoeckol-A (**241**), which selectively inhibited BChE (IC_50_ = 0.9 ± 0.2 µM), and phlorotannin 974-B (**242**) that acted as an effective dual inhibitor (IC_50AChE_ = 1.95 ± 0.01 µM; IC_50BChE_ = 3.26 ± 0.08 µM) (Figs. **22** and **23**). Also, two terpenoids obtained from the brown alga *Sargassum siliquastrum* (Sargassaceae), known as sargachromanol I (**245**) and sargachromanol G (**246**), were identified as potent reversible AChE inhibitors among 640 natural compounds examined by Lee and co-workers (Fig. **23**) [67].

From Table **15**, which lists the active compounds isolated from animals during the last decade, the results observed for compounds **247**, **252** and **257** are remarkable. Bufalin (**247**), obtained from the venom of *Bufo bufo gargarizans,* was the most potent AChEi with IC_50_ *=* 0.12 µM and moderate toxicity in brine shrimp assay [68]. A few references can be found in the literature about the activity of skin extracts from amphibian anuran (frogs and toads) as a source of bioactive compounds, among which peptides act as inhibitors of AChE, BChE and MAO-B enzymes [69, 70]. Compared to those peptides, compound **247** is the most effective AChEi identified for amphibians.

Three brominated alkaloids isolated from sponges stand out in Table **15** due to their low micromolar IC_50_ values for AChE: discorhabdin G (**254**), one of the alkaloids found in *Latrunculia biformis* [71] and purealidin Q (**259**) and aplysamine 2 (**260**), obtained from *Pseudoceratina* cf. *purpurea* (Figs. **24** and **25**) [72].

Finally, the best AChEi in Table **15** is asperdiol (**257**), a diterpene cembranoid isolated from *Eunicea knighti,* a sea whip collected in the Caribbean Sea, with IC_50_ = 0.358 ± 0.130 µM (Fig. **25**) [73].

## CONCLUSION

More than a century ago, Alois Alzheimer described the symptoms of what would be known later as Alzheimer's disease, a neurodegenerative disorder for which science has not yet found a cure or a way of preventing it. The aging of the world's population, together with the fact that the prevalence of this disease increases in elderly people, make the search for an effective treatment a relevant issue still to be addressed. The development of the “cholinergic hypothesis” as the main therapeutic approach in the treatment of AD, has pushed the discovery of new and effective molecules with the ability to inhibit cholinesterase enzymes. Despite the enormous amount of research on this topic, the exploration of natural products with cholinesterase inhibitory activity is still a matter of interest for the scientific global community. On the other hand, as it is shown in this work, Nature continues to be an important source of secondary metabolites with outstanding biological activities. This review analyzes 142 selected studies published during the period 2012-2022, about natural compounds with the ability to inhibit AChE and/or BChE, including 260 active compounds. This work intends to be a tool for those interested in the topic as well as for those looking for inspiration for the design and synthesis of new bioactive molecular entities.

## Figures and Tables

**Fig. (19):**
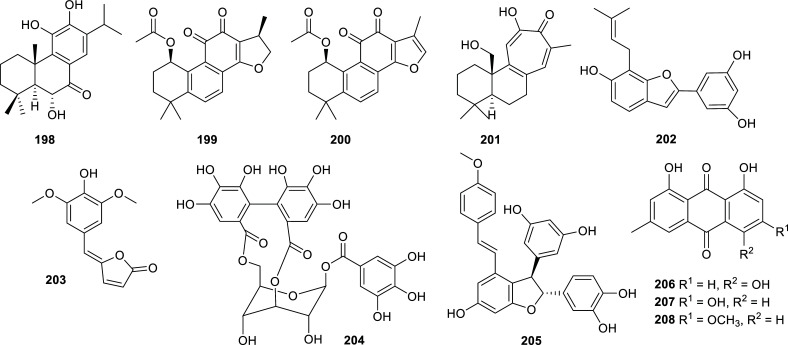
Compounds reported as potent cholinesterase inhibitors isolated from plants of Lamiaceae Moraceae Orchidaceae, Phyllanthaceae, and Polygonaceae families.

**Fig. (20):**
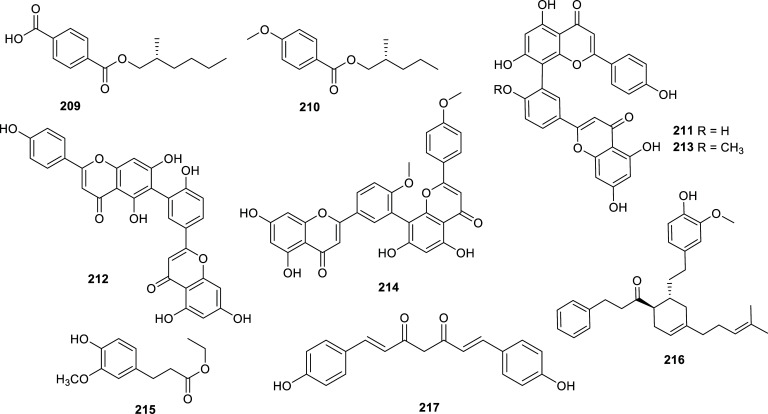
Compounds reported as potent cholinesterase inhibitors isolated from plants of Polygonaceae (con.), Roseaceae, Selaginellaceae, Solanaceae and Zingiberaceae.

**Table 1:** Potent cholinesterase inhibitors isolated from plants of Amaryllidaceae and Lycopodiaceae families reported in 2012-2022.

**Family**	**Species**	**Compound Name**	**Compound Class**	**AChE^a^** **IC_50_ ± SD^c^**	**BChE^b^** **IC_50_ ± SD^c^**	**References**
Amaryllidaceae	*Amaryllis belladonna*	Acetylcaranine (**1**)	Isoquinoline alkaloid	11.7 ± 0.7	n.d.	[74]
	*Narcissus * *pseudonarcissus*	Narciabduliine (**2**)	Narcikachnine alkaloid	3.29 ± 0.73^d^	3.44 ± 0.02^e^	[27]
	*Zephyranthes candida*	Zephycandidine III (**3**)	Phenanthridine alkaloid	8.82	n.d.	[75]
	*Zephyranthes citrina*	Narcieliine (**4**)	Narcikachnine alkaloid	18.7 ± 2.3^d^	1.34 ± 0.31^e^	[28]
Lycopodiaceae	*Huperzia carrinata*	8,15-dihydrohuperzine A (**5**)	Lycodine-type akaloid	2.63 ± 0.37	n.d.	[29]
		12-epilycodine *N*-oxide (**6**)	Lycopodine-type alkaloid	0.59 ± 0.06	n.d.	[29]
		Lycodoline (**7**)	Lycopodine-type alkaloid	13.6 ± 0.78	n.d.	[29]
		Gnidioidine (**8**)	Lycopodine-type alkaloid	3.44 ± 0.20	n.d.	[29]
		Lycoposerramine K (**9**)	Lycopodine-type alkaloid	11.6 ± 0.60	n.d.	[29]
	*Huperzia squarrosa*	Squarrosine A (**10**)	Alkaloid	7.30 ± 0.12	n.d.	[76]
		Pyrrolhuperzine A (**11**)	Alkaloid	8.91 ± 0.83	n.d.	[76]
	*Lycopodiastrum * *casuarinoides*	Casuarinine I (**12**)	Lycodine-type alkaloid	12.1	n.d.	[30]
		*N*-demethylhuperzinine (**13**)	Lycodine-type alkaloid	15.0	n.d.	[30]
		Huperzine C (**14**)	Lycodine-type alkaloid	0.489	n.d.	[30]
	*Lycopodiella cernua*	21β-hydroxyserrat-14-en-3,16-dione (**15**)	Serratene-type triterpenoid	10.67 ± 0.66	> 30	[31]
		3β,14α,15α,21β-tetrahydroxyserratan-24-oic acid-3β-yl-(4ʹ-methoxy-5ʹ hydroxybenzoate) (**16**)	Serratene-type triterpenoid	9.98 ± 0.29	> 30	[31]
		(2E,4E,6R)-6-hydroxydeca-2,4-dienoic acid (**17**)	Unsaturated fatty acid	0.22 ± 0.03	> 30	[31]
	*Phlegmariurus henryi*	Phleghenrine A (**18**)	Alkaloid	4.91 ^d^	> 30 ^e^	[77]
		Phleghenrine D (**19**)	Alkaloid	4.32 ^d^	> 100 ^e^	[77]

**Note:** ^a^AChE from *Electrophorus electricus* unless otherwise stated. ^b^BChE from equine serum unless otherwise stated. ^c^IC_50_ values are expressed in µM, SD values are included when available, n.d. not determined. ^d^*h*AChE, ^e^*h*BChE.

**Table 2:** Alkaloids with potent cholinesterase inhibition isolated from plants of Menispermaceae family reported in 2012-2022.

**Species**	**Compound Name**	**Alkaloid Type**	**AChE^a^** **IC_50_ ± SD^c^**	**BChE^b^** **IC_50_ ± SD^c^**	**References**
*Cissampelos pareira*	(-)-cyclanoline (**20**)	Protoberberine	4.62 ± 0.18	n.d.	[78]
	(+)-obamegine (**21**)	Bisbenzylisoquinoline	3.26 ± 0.22	n.d.	[78]
	(+)-homoaromoline (**22**)	Bisbenzylisoquinoline	4.86 ± 0.29	n.d.	[78]
	(-)-nor-*N*׳-chondrocurine (**23**)	Bisbenzylisoquinoline	7.46 ± 0.19	n.d.	[78]
*Stephania Epigaea*	Epiganine B (**24**)	Aporphine	4.36	n.d.	[79]
	Dehydrodicentrine (**25**)	Aporphine	2.98	5.57 ± 0.15	[32, 79]
	Romerine (**26**)	Aporphine	8.32	n.d.	[79]
	Romeline (**27**)	Aporphine	13.9	n.d.	[79]
	Phanostenine (**28**)	Aporphine	15.5	n.d.	[79]
	Dicentrine (**29**)	Aporphine	6.6	n.d.	[79]
*Stephania pierrei*	(-)-stephanine (**30**)	Aporphine	11.34 ± 0.20	2.80 ± 0.07	[32]
	Dehydroroemerine (**31**)	Aporphine	1.21 ± 0.09	3.34 ± 0.02	[32]
	Dehydrostephanine (**32**)	Aporphine	2.85 ± 0.24	3.26 ± 0.05	[32]
*Stephania tetrandra*	Fangchinoline (**33**)	Bisbenzylisoquinoline	2.58 ± 0.28^d^	n.i.^e^	[80]

**Note:** ^a^AChE from *Electrophorus electricus* unless otherwise stated. ^b^BChE from equine serum unless otherwise stated. ^c^IC_50_ values are expressed in µM, SD values are included when available, n.d. not determined, n.i. no inhibition. ^d^mouse brain AChE. ^e^mouse brain BChE.

**Table 3:** Alkaloids with potent cholinesterase inhibition isolated from plants of Papaveraceae family reported in 2012-2022.

**Species**	**Compound Name**	**Alkaloid Type**	**AChE^a^** **IC_50_ ± SD^c^**	**BChE^b^** **IC_50_ ± SD^c^**	**References**
*Chelidonium majus*	chelerythrine (**34**)	Isoquinoline	1.54 ± 0.07^e^	6.33 ± 0.93	[34]
*Papaver setiferum*	7,8-didehydromecambridine (**35**)^d^	Protoberberine	10.3 ± 1.1	> 100	[35]
	7,8-didehydroorientalidine (**36**)^d^	Protoberberine	3.4 ± 4.7	> 50	[35]
	alborine (**37**)	Protoberberine	6.8 ± 4.5	> 50	[35]
	orientalidine (**38**)	Protoberberine	5.0 ± 1.0	> 100	[35]
	*N*-methylisothebainium (**39**)	Protoberberine	n.i.	7.1 ± 2.7	[35]

**Note:** ^a^AChE from *Electrophorus electricus* unless otherwise stated. ^b^BChE from equine serum. ^c^IC_50_ values are expressed in µM, SD values are included when available, n.i. no inhibition. ^d^isolated as TFA salt. ^e^*h*AChE.

**Table 4:** Alkaloids with potent cholinesterase inhibition isolated from plants of Ranunculaceae family reported in 2012-2022.

**Species**	**Compound Name**	**Alkaloid Type**	**AChE^a^** **IC_50_ ± SD^c^**	**BChE^b^** **IC_50_ ± SD^c^**	**References**
*Aconitum * *heterophyllum*	6β-methoxy-9β-dihydroxylheteratisine (**40**)	Diterpenoid-alkaloid	5.4 ± 0.01	8.6 ± 0.27	[81]
	1α,11,13β-trihydroxylhetisine (**41**)	Diterpenoid-alkaloid	6.5 ± 0.09	9.3 ± 0.73	[81]
	6,15β-dihydroxylhetisine (**42**)	Diterpenoid-alkaloid	12.8 ± 0.11	16.2 ± 0.4	[81]
	Iso-atisine (**43**)	Diterpenoid-alkaloid	8.4 ± 0.51	13.1 ± 0.4	[81]
	Heteratisine (**44**)	Diterpenoid-alkaloid	6.5 ± 0.21	10.4 ± 0.15	[81]
	Hetisinone (**45**)	Diterpenoid-alkaloid	10.1 ± 1.30	15.6 ± 0.88	[81]
	19-epiisoatisine (**46**)	Diterpenoid-alkaloid	10.3 ± 0.13	14.7 ± 0.35	[81]
	Atidine (**47**)	Diterpenoid-alkaloid	14.6 ± 0.53	18.1 ± 0.07	[81]
*Cimicifuga dahurica*	(*E*)-3-(3´-methyl-2´-butenylidene)-1-methyl-2-indolinone (**48**)	Indolinone	> 50	13.8 ± 1.5	[82]
	(*E*)-3-(3´-methyl-2´-butenylidene)-2-indolinone (**49**)	Indolinone	> 50	6.5 ± 2.5	[82]
*Delphinium denudatum*	Jadwarine-A (**50**)	Norditerpenoid-alkaloid	9.2 ± 0.12	19.6 ± 0.72	[83]
	Isotalatizidine hydrate (**51**)	Diterpenoid-alkaloid	12.1 ± 0.43	21.4 ± 0.23	[83]
	Dihydropentagynine (**52**)	Diterpenoid-alkaloid	11.2 ± 0.23	22.2 ± 0.33	[83]
*Dichocarpum auriculatum*	Dauricine (**53**)	Bisbenzylisoquinoline	1.41 ± 0.02	n.d.	[37]

**Note:** ^a^AChE from *Electrophorus electricus.*^b^BChE from equine serum. ^c^IC_50_ values are expressed in µM, SD values are included when available, n.d. not determined.

**Table 5:** Alkaloids with potent cholinesterase inhibition isolated from plants of Apocynaceae and Liliaceae families reported in 2012-2022.

**Family**	**Species**	**Compound Name**	**Alkaloid Type**	**AChE^a^** **IC_50_ ± SD^c^**	**BChE^b^** **IC_50_ ± SD^c^**	**References**
Apocynaceae	*Geissospermum vellosii*	Geissoschizoline (**54**)	Indole	5.86 ± 0.31	7.89 ± 0.33	[38]
		Geissoschizone (**55**)	Indole	8.50 ± 0.43	11.46 ± 0.44	[38]
		3′,4′,5′,6′-tetradehydrogeissospermine (**56**)	Indole	0.45 ± 0.01	0.32 ± 0.02	[38]
	*Holarrhena pubescens*	Mokluangin A (**57**)	Steroidal	2.12 ± 0.06	n.d.	[84]
		Mokluangin C (**58**)	Steroidal	1.44 ± 0.66	n.d.	[84]
		Antidysentericine (**59**)	Steroidal	4.09 ± 0.05	n.d.	[84]
	*Rauvolfia reflexa*	Isoresrpiline (**60**)	Indole	24.89	11.96	[85]
		Rescinnamine (**61**)	Indole	11.01	8.06	[85]
	*Rauwolfia serpentina*	Ajmalicine (**62**)	Indole	3.5 ± 1.41	5.44 ± 1.75	[86]
	*Rauwolfia* spp.	Rauwolscine (**63**)	Indole	n.i.	8.38 ± 0.54	[34]
	*Vinca rosea*	Catharanthine (**64**)	Indole	n.i.	5.17 ± 0.18	[34]
Liliaceae	*Fritillaria walujewii*	Walujewine A (**65**)	Isosteroidal	7.60 ± 0.51	> 100	[40]
		Tortifoline (**66**)	Isosteroidal	5.85 ± 0.63	2.08 ± 0.50	[40]
		Tortifoline glucoside (**67**)	Isosteroidal	10.75 ± 1.25	3.89 ± 0.74	[40]
		Walujewine C (**68**)	Isosteroidal	7.19 ± 0.32	2.58 ± 0.27	[40]
		Walujewine D (**69**)	Isosteroidal	> 50	13.50 ± 0.84	[40]
		Sinpeinine A (**70**)	Isosteroidal	8.37 ± 1.41	3.05 ± 0.33	[40]
		Hepehenizioiside (**71**)	Isosteroidal	11.46 ± 1.18	6.80 ± 0.63	[40]
		Walujewine E (**72**)	Isosteroidal	9.88 ± 1.45	5.71 ± 0.48	[40]

**Note:** ^a^AChE from *Electrophorus electricus.*^b^BChE from equine serum. ^c^IC_50_ values are expressed in µM. SD values are included when available, n.d. not determined, n.i. no inhibition.

**Table 6:** Potent cholinesterase inhibitors isolated from plants of Rubiaceae family reported in 2012-2022.

**Species**	**Compound name**	**Compound Class**	**AChE^a^** **IC_50_ ± SD^c^**	**BChE^b^** **IC_50_ ± SD^c^**	**References**
*Hedyotis * *diffusa*	6-*o*-methylscandoside methyl ester (**73**)	Iridoid glycoside	n.i.	11.59 ± 0.68	[87]
	quercetin-3-*O*-[2”-*O*-(6”’-*O*-E-feruloyl)-β-D-glucopyranosyl]-β-D-glucopyranoside (**74**)	Flavonol glycoside	˃30	13.77 ± 0.37	[87]
*Nauclea * *officinalis*	angustidine (**75**)	Monoterpene-indole alkaloid	21.72	1.03	[42]
	nauclefine (**76**)	Monoterpene-indole alkaloid	n.d.	7.70	[42]
	angustine (**77**)	Monoterpene-indole alkaloid	n.d.	4.98	[42]
	harmane (**78**)	Monoterpene-indole alkaloid	˃100	13.18	[42]
*Uncaria * *rhynchophlly*	7-epi-javaniside (**79**)	Monoterpene-indole alkaloid	2.85 ± 0.50	2.13 ± 0.10	[43]
	vincosamide (**80**)	Monoterpene-indole alkaloid	12.4 ± 0.86	23.18 ± 0.14	[43]
	strictosamide (**81**)	Monoterpene-indole alkaloid	˃30	6.47 ± 0.72	[43]
*Uncaria* spp.	hirsutine (**82**)	Indole alkaloid	n.i.	4.97 ± 0.33	[34]

**Note:** ^a^AChE from *Electrophorus electricus.*^b^BChE from equine serum. ^c^IC_50_ values are expressed in µM. SD values are included when available, n.d. not determined, n.i. no inhibition.

**Table 7:** Potent AChE inhibitors isolated from plants of Theaceae family reported in 2012-2022.

**Species**	**Compound Name**	**Compound Class**	**AChE^a^** **IC_50_ ± SD**	**References**
*Camellia sinensis*	3-*O*-cinnamoylepicatechin (**83**)	Flavan-3-ol	1.04 ± 0.13	[45]
	(−)-6-(5′′′*S*)-*N*-ethyl-2-pyrrolidinone-3-O-cinnamoylepicatechin (**84**)	Flavoalkaloid	0.14 ± 0.09	[45]
	(−)-6-(5′′′*R*)-*N*-ethyl-2-pyrrolidinone-3-*O*-cinnamoylepicatechin (**85**)	Flavoalkaloid	0.13 ± 0.02	[45]
	(−)-8-(5′′′*S*)-*N*-ethyl-2-pyrrolidinone-3-*O*-cinnamoylepicatechin (**86**)	Flavoalkaloid	0.16 ± 0.02	[45]
	(−)-8-(5′′′*R*)-*N*-ethyl-2-pyrrolidinone-3-*O*-cinnamoylepicatechin (**87**)	Flavoalkaloid	0.21 ± 0.04	[45]
*Camellia sinensis* var. *assamica*	(−)-8-(5′′*R/S*)-*N*-ethyl-2-pyrrolidinone-epiafzelechin-3-*O*-gallate (**88**)	Flavoalkaloid	14.22 ± 0.57^b^	[88]
	(−)-epiafzelechin-3- *O*-gallate (**89**)	Flavoalkaloid	10.81 ± 0.13^b^	[88]
	(-)-epicatechin 3-*O*-caffeoate (**90**)	Catechin	2.49 ± 0.43	[89]
	Epigallocatechin 3-*O*-p-coumaroate (**91**)	Catechin	11.41 ± 2.00	[89]
*Camellia sinensis var.pubilimba*	Ethylpyrrolidinonyl epi-catechin-3-*O*-gallate (**92**)	Flavan-3-ol	1.97 ± 0.06^b^	[90]
	5′′*R*-ethylpyrrolidinonyl epigallocatechin-3-O-gallate (**93**)	Flavan-3-ol	1.93 ± 0.13^b^	[90]
	5′′*R*-ethylpyrrolidinonyl epicatechin (**94**)	Flavan-3-ol	9.81 ± 0.93^b^	[90]
	5′′*R*-ethylpyrrolidinonyl catechin (**95**)	Flavan-3-ol	2.13 ± 0.12^b^	[90]

**Note:** ^a^AChE from *Electrophorus electricus,* IC_50_ values are expressed in µM, SD values are included when available, n.d. not determined. ^b^*h*AChE.

**Table 8:** Potent cholinesterase inhibitors isolated from plants of Apiaceae family reported in 2012-2022.

**Species**	**Compound Name**	**Compound Class**	**AChE^a^** **IC_50_ ± SD^c^**	**BChE^b^** **IC_50_ ± SD^c^**	**References**
*Angelica polymorpha*	Phellopterin (**96**)	Coumarin	4.0	n.d.	[91]
	Xanthyletin (**97**)	Coumarin	8.8	n.d.	[91]
	Cnidilin (**98**)	Coumarin	6.3	n.d.	[91]
	Geijerine (**99**)	Coumarin	9.7	n.d.	[91]
	(−)-3'-acetyl hamaudol (**100**)	Chromone	12.6	n.d.	[91]
	7-demethylsuberosine (**101**)	Coumarin	8.7	n.d.	[91]
	Dehydrogeijerin (**102**)	Coumarin	10.4	n.d.	[91]
	(+)-visamminol (**103**)	Chromone	8.7	n.d.	[91]
	Divaricatol (**104**)	Chromone	12.9	n.d.	[91]
*Ferula gummosa*	Kellerin (**105**)	Hydroxycoumarin	10.6	n.d.	[92]
*Ferula lutea*	Psoralen (**106**)	Furanocoumarin	6.48 ± 1.05	n.d.	[49]
	Xanthoxin (**107**)	Furanocoumarin	0.76 ± 0.3	n.d.	[49]
*Heptaptera cilicica*	Umbelliprenin (**108**)	Sesquiterpene coumarin ether	5.86 ± 0.03	1.10 ± 0.19	[50]
	Umbelliprenin-10′,11′-monoepoxide (**109**)	Sesquiterpene coumarin ether	> 100	12.59 ± 0.02	[50]
	Conferone (**110**)	Sesquiterpene coumarin ether	3.31 ± 0.01	9.31 ± 0.28	[50]
	Mogoltacin (**111**)	Sesquiterpene coumarin ether	1.95 ± 0.05	9.74 ± 0.01	[50]
	Feselol (**112**)	Sesquiterpene coumarin ether	1.26 ± 0.01	9.98 ± 0.24	[50]
*Heracleum * *moellendorffii*	Angelicin (**113**)	Coumarin	10.2	n.d.	[93]

**Note:** ^a^AChE from *Electrophorus electricus.*^b^BChE from equine serum. ^c^IC_50_ values are expressed in µM, SD values are included when available, n.d. not determined.

**Table 9:** Potent cholinesterase inhibitors isolated from plants of Myristicaceae and Myrtaceae families reported in 2012-2022.

**Family**	**Species**	**Compound Name**	**Compound Class**	**AChE^a^** **IC_50_ ± SD^c^**	**BChE^b^** **IC_50_ ± SD^c^**	**References**
Myristicaceae	*Knema pachycarpa*	Knepachycarpic acid A (**114**)	Anacardic acid derivative	8.19 ± 0.63	n.d.	[94]
		Knepachycarpic acid B (**115**)	Anacardic acid derivative	3.89 ± 0.33	n.d.	[94]
		Knepachycarpanol A (**116**)	Cardanol derivative	2.60 ± 0.24	n.d.	[94]
		Knepachycarpanol B (**117**)	Cardanol derivative	7.09 ± 0.59	n.d.	[94]
		Knepachycarpasinol (**118**)	Cardol derivative	2.46 ± 0.23	n.d.	[94]
	*Myristica cinnamomea*	Malabaricone E (**119**)	Acyl phenol	6.44 ± 0.85	6.65 ± 0.13	[51]
		Malabaricone A (**120**)	Acyl phenol	1.31 ± 0.17	>30	[51]
		Malabaricone B (**121**)	Acyl phenol	1.84 ± 0.19	1.76 ± 0.21	[51]
		Malabaricone C (**122**)	Acyl phenol	1.94 ± 0.27	2.80 ± 0.4	[51]
		Maingayone A (**123**)	Acyl phenol	12.66 ± 1.48	10.51 ± 2.07	[51]
		Maingayone B (**124**)	Acyl phenol	>30	12.52 ± 2.86	[51]
	*Myristica fragrans*	Macelignan (**125**)	Lignan	4.16 ± 0.07	9.69 ± 0.98	[67]
Myrtaceae	*Callistemon salignus*	Callistemontrimer A (**126**)	Acylphloroglucinol trimer	11.2 ± 1.01^d^	n.d.	[95]
		Callistemontrimer B (**127**)	Acylphloroglucinol trimer	7.45 ± 0.81^d^	n.d.	[95]
		Callistemontrimer C (**128**)	Acylphloroglucinol trimer	2.28 ± 0.19^d^	n.d.	[95]
		Callistemontrimer D (**129**)	Acylphloroglucinol trimer	4.96 ± 0.39^d^	n.d.	[95]
Myrtaceae	*Eucalyptus robusta*	(±)-eucalyprobusal F (**130**)	Phloroglucinol-monoterpene	3.22 ± 0.36^d^	n.d.	[96]
		(±)-eucalyprobusone C (**131**)	Dimeric-acylphloroglucinol	3.82 ± 0.22	n.d.	[96]
	*Rhodomyrtus tomentosa*	Rhotomentosone E (**132**)	Polycyclic phloroglucinol	8.68 ± 1.12	n.d.	[97]
	*Syzygium jambos*	6-heptadeca-8*Z*,11*Z*,14*Z*-trienyl salicylic acid (**133**)	Anacardic acid derivative	2.4	n.d.	[52]
		Anacardic acid C (**134**)	Anacardic acid derivative	0.54	n.d.	[52]

**Note:** ^a^AChE from *Electrophorus electricus.* unless otherwise stated ^b^BChE from equine serum. ^c^IC_50_ values are expressed in µM, SD values are included when available, n.d. not determined. ^d^*h*AChE.

**Table 10:** Potent cholinesterase inhibitors isolated from plants of Clusiaceae family reported in 2012-2022.

**Species**	**Compound Name**	**Compound Class**	**AChE^a^** **IC_50_ ± SD^c^**	**BChE^b^** **IC_50_ ± SD^c^**	**References**
*Garcinia hombroniana*	2β-hydroxy-3α-O-caffeoyltaraxar-14-en-28-oic acid (**135**)	Triterpenoid	13.5 ± 0.95	10.6 ± 0.54	[98]
	Garcihombronane N (**136**)	Triterpenoid	17.5	10.4	[99]
	2,3’,4,5’-tetrahydroxy-6-methoxybenzophenone (**137**)	Polyphenol	10.3	>30	[100]
*Garcinia mangostana*	Mangostanol (**138**)	Xanthone	5.77	10.41	[54]
	3-isomangostin (**139**)	Xanthone	5.75	12.96	[54]
	α-mangostin (**140**)	Xanthone	2.14	5.41	[54]
	γ-mangostin (**141**)	Xanthone	1.31	1.78	[54]
	8-deoxygartanin (**142**)	Xanthone	20.41	6.47	[54]
	Garcinone C (**143**)	Xanthone	1.24	8.96	[54]
*Hypericum uralum*	Hyperuralone C (**144**)	Acylphloroglucinol	9.6 ^d^	n.d.	[101]
	Hyperuralone D (**145**)	Acylphloroglucinol	7.1 ^d^	n.d.	[101]

**Note:** ^a^AChE from *Electrophorus electricus* unless otherwise stated. ^b^BChE from equine serum. ^c^IC_50_ values are expressed in µM, SD values are included when available, n.d. not determined. ^d^*h*AChE.

**Table 11:** Potent cholinesterase inhibitors isolated from plants of Scrophulariaceae families reported in 2012-2022.

**Species**	**Compound name**	**Compound Class**	**AChE^a^** **IC_50_ ± SD^c^**	**BChE^b^** **IC_50_ ± SD^c^**	**References**
*Buddleja asiatica*	Asiatoate A (**146**)	Benzoate	5.54	>30	[102]
	Asiatoate B (**147**)	Benzoate	8.34	>30	[102]
*Leucophyllum * *ambiguum*	(3*S*)-hydroxy-3′,4′-dimethoxy-L-phenylalanine (**148**)	α-amino acid	0.001	n.d.	[55]
	Ciquitin A (**149**)	Furofuranone lignan	0.003	n.d.	[55]
	Ciquitin B (**150**)	Furofuranone lignan	0.028	n.d.	[55]
	Ciquitin C (**151**)	Furofuranone lignan	2.229	n.d.	[55]
	Ciquitin D (**152**)	Furofuranone lignan	0.158	n.d.	[55]

**Note:** ^a^AChE from *Electrophorus electricus.*^b^BChE from equine serum. ^c^IC_50_ values are expressed in µM, SD values are included when available, n.d. not determined.

**Table 12:** Alkaloids with potent cholinesterase inhibition isolated from plants of different families reported in 2012-2022.

**Family**	**Species**	**Compound Name**	**Alkaloid Type**	**AChE^a^** **IC_50_ ± SD^c^**	**BChE^b^** **IC_50_ ± SD^c^**	**References**
Berberidaceae	*Berberis aquifolium*	Oxyacanthine sulfate (**153**)	Isoquinoline	n.i.	4.10 ± 0.20	[34]
	*Epimedium koreanum*	Epimediphine (**154**)	Aporphine	3.1	n.d.	[103]
Caprifoliaceae	*Valeriana officinalis* var. *latifolia*	Valerianofal A (**155**)	Sesquiterpenoid-alkaloid	>100 µg/mL	2.8 µg/mL	[104]
		Actinidine (**156**)	Pyridine	20.9 µg/mL	0.4 µg/mL	[104]
Euphorbiaceae	*Croton heliotropiifolius*	(S)-magnoflorine (**157**)	Aporphine	13.2 ± 0.8	n.d.	[105]
		(+)-menisperine (**158**)	Aporphine	14.3 ± 0.5	n.d.	[105]
		Taspine (**159**)	Bislactone amine	8.5 ± 0.3	n.d.	[105]
		Moschamine (**160**)	Indole	9.2 ± 0.4	n.d.	[105]
Fumariaceae	*Corydalis saxicola*	(−)-2,9-dihydroxyl-3,11-dimethoxy-1,10-dinitrotetrahydroprotoberberine (**161**)	Isoquinoline	8.77 ± 0.20	n.d.	[106]
		(+)-1-nitroapocavidine (**162**)	Isoquinoline	1.70 ± 0.31	n.d.	[106]
		Dehydrocavidine (**163**)	Isoquinoline	9.92 ± 0.23	n.d.	[106]
Lauraceae	*Cryptocarya infectoria*	2-methoxyatherosperminine (**164**)	Amino-phenanthrene	n.d.	3.95	[107]
Magnoliaceae	*Magnolia biondii* Pamp.	4,4'-dihydroxy-3-methoxy-paucine-4'-*O*-β-D-glucopyranoside (**165**)	Ferulic acid amide	12.5 ± 2.5	n.d.	[108]
		(+)-reticuline *N*-oxide (**166**)	Benzylisoquinoline	10.4 ± 2.4	n.d.	[108]
		(+)-isococlaurine (**167**)	Benzylisoquinoline	8.2 ± 1.8	n.d.	[108]
Malphighiaceae	*Tetrapterys mucronata*	Bufotenine (**168**)	Tryptamine	11.4 ± 0.2	n.d.	[109]
		5-methoxy-*N*-methyltryptamine (**169**)	Tryptamine	12.5 ± 0.3	n.d.	[109]
		5-methoxy-*N,N*-dimethyltryptamine (**170**)	Tryptamine	14.0 ± 0.2	n.d.	[109]
		Mucronatin A (**171**)	Bisindole	11.7 ± 0.4	n.d.	[109]
		Mucronatin B (**172**)	Bisindole	12.7 ± 0.3	n.d.	[109]
Melanthiaceae	*Veratrum álbum, V. viride*	Veratramine (**173**)	Steroidal	n.i.	10.78 ± 1.13	[34]
Nelumbonaceae	*Nelumbo nucifera*	Neferine (**174**)	Bisbenzylisoquinoline	14.19 ± 1.46	˃30	[56]
		Liensinine (**175**)	Bisbenzylisoquinoline	0.34 ± 0.02	9.96 ± 0.47	[56]
Nitrariaceae	*Peganum harmala*	Peganole (**176**)	Quinazoline	n.i.	11.39 ± 0.37	[34]
		Vasicine (**177**)	Quinazoline	n.i.	2.53 ± 0.36	[34]
	*Nitraria schoberi*	Nitrarine (**178**)	Indole	n.i.	10.63 ± 0.37	[34]
Rutaceae	*Ravenia spectabilis*	Arborinine (**179**)	Acridone	13.14 ± 0.07	26.34 ± 0.31	[110]
	*Zanthoxylum rigidum*	Nitidine (**180**)	Benzophenanthridine	0.65 ± 0.09	5.73 ± 0.60	[58]
		Avicine (**181**)	Benzophenanthridine	0.15 ± 0.01	0.88 ± 0.08	[58]
Stemonaceae	*Stichoneuron caudatum*	Sessilistemonamine E (**182**)	Stichoneurine-type	9.1 ± 0.15^d^	n.d.	[111]
Zygophyllaceae	*Peganum harmala Linn*	(1*S*)2-aldehyde-tetrahydroharmine (**183**)	Harmine	12.35 ± 0.24	5.51 ± 0.33	[112]

**Note:** ^a^AChE from *Electrophorus electricus* unless otherwise stated. ^b^BChE from equine serum. ^c^IC_50_ values are expressed in µM, unless otherwise stated. SD values are included when available, n.d. not determined, n.i. no inhibition. ^d^*h*AChE.

**Table 13:** Other compounds reported as potent cholinesterase inhibitors isolated from plants in 2012-2022.

**Family**	**Species**	**Compound Name**	**Compound Class**	**AChE^a^** **IC_50_ ± SD^c^**	**BChE^b^** **IC_50_ ± SD^c^**	**References**
Asteraceae	*Achillea biebersteinii*	Quercetin-7-*O*-β-D-glucoside (**184**)	Flavonoid glycoside	1.84 ± 0.07	2.24 ± 0.25	[113]
		Patuletin-7-*O*-β-D-glucoside (**185**)	Flavonoid glycoside	1.77 ± 0.05	2.27 ± 0.06	[113]
Asteraceae	*Volutaria abyssinica*	Amberboin (**186**)	Sesquiterpene lactone	0.79 ± 0.03	0.58 ± 0.13	[59]
		Lipidiol (**187**)	Sesquiterpene lactone	0.52 ± 0.01	n.i.	[59]
Cornaceae	*Cornus officinalis*	7-*O*-galloyl-D-sedoheptulose (**188**)	Polyphenol	10.50 ± 1.16	>50	[60]
		Tellimagrandin II (**189**)	Gallotannin	11.86 ± 0.56	18.29 ± 0.01	[114]
		Loganin (**190**)	Iridoid glycoside	0.33 ± 0.05	>30	[60]
		Morroniside (**191**)	Iridoid glycoside	3.95 ± 0.17	>30	[60]
Fabaceae	*Dalea elegans*	Glabranin (**192**)	Flavanone	0.006 ± 0.001^d^	n.d.	[62]
	*Cullen corylifolium*	Neobavaisoflavone (**193**)	Isoflavonoid	0.003	0,076	[115]
	*Glycyrrhiza uralensis*	Glycyrol (**194**)	Coumarin	14.77 ± 0.19	7.22 ± 0.37	[116]
		7-*O*-methylluteone (**195**)	Flavonoid	9.9	n.d.	[117]
Hypericaceae	*Hypericum perforatum*	Hypericin (**196**)	Naphthodianthrone	11.3	n.d.	[118]
Juglandaceae	*Juglans regia*	1-hydroxy-5,5-dimethyl-5,6,7,8-tetrahydro-9,10-anthraquinone (**197**)	Anthraquinone	7.79	n.d.	[119]
Lamiaceae	*Caryopteris mongolica*	6α-hydroxydemethylcryptojaponol (**198**)	Abietane diterpenoid	12.3 ± 3.0	7.70 ± 0.09	[120]
	*Perovskia atriplicifolia*	(1*R*,15*R*)-1-acetoxycryptotanshinone (**199**)	Norditerpenoid	n.i.	2.37	[121]
		(1*R*)-1-acetoxytanshinone IIA (**200**)	Norditerpenoid	n.i.	7.86	[121]
		Isograndifoliol (**201**)	Norditerpenoid	>100	0.89	[121]
Moraceae	*Morus radix*	Moracin S (**202**)	Benzofuran derivative	>30	7.22 ± 0.22	[122]
Orchidaceae	*Goodyera * *schlechtendaliana*	Goodyschle A (**203**)	Butenolide	>50	6.88 ± 1.63	[123]
Phyllanthaceae	*Phyllanthus niruri*	Isocorilagin (**204**)	Tannin	0.31 μg/mL	2.66 μg/mL	[124]
Polygonaceae	*Rheum lhasaense*	4′-methoxy-scirpusin A (**205**)	Stilbenoid	2.18 ± 0.67	n.d.	[125]
	*Rumex abyssinicus*	Helminthosporin (**206**)	Anthraquinone	2.63 ± 0.09	2.99 ± 0.55	[126]
		Emodin (**207**)	Anthraquinone	15.21 ± 3.52	n.d.	[126]
		Physcion (**208**)	Anthraquinone	12.16 ± 0.36	n.d.	[126]
Roseaceae	*Cotoneaster * *horizontalis*	Horizontoate A (**209**)	Aromatic ester	1.54 ± 0.03	5.97 ± 0.08	[127]
		Horizontoate B (**210**)	Aromatic ester	3.41 ± 0.02	6.84 ± 0.06	[127]
Selaginellaceae	*Selaginella * *doederleinii*	Amentoflavone (**211**)	Biflavonoid	0.73 ± 0.01	n.d.	[128]
		Robustaflavone (**212**)	Biflavonoid	6.16 ± 0.03	n.d.	[128]
		Bilobetin (**213**)	Biflavonoid	5.76 ± 0.02	n.d.	[128]
		Isoginkgetin (**214**)	Biflavonoid	4.11 ± 0.02	n.d.	[128]
Solanaceae	*Lycium barbarum*	Ethyl dihydroferulate (**215**)	Polyphenol	11	n.d.	[129]
Zingiberaceae	*Alpinia officinarum*	(-)-alpininoid B (**216**)	Diarylheptanoid	2.6 ± 4.2	n.d.	[130]
	*Curcuma longa* L.	Bisdemethoxycurcumin (**217**)	Curcuminoid	2.14 ± 0.78	>50	[131]

**Note:** ^a^AChE from *Electrophorus electricus* unless otherwise stated. ^b^BChE from equine serum. ^c^IC_50_ values are expressed in µM, unless otherwise stated, SD values are included when available, n.d. not determined, n.i. no inhibition. ^d^*h*AChE.

**Table 14:** Potent cholinesterase inhibitors isolated from fungi and algae reported in 2012-2022.

-	**Family**	**Species**	**Compound Name**	**Compound Class**	**AChE^a^** **IC_50_ ± SD^c^**	**BChE^b^** **IC_50_ ± SD^c^**	**References**
Fungi	Hypoxylaceae	*Daldinia* sp.	Daldiniol A (**218**)	Acetylenic benzyl alcohol	n.d.	6.93 ± 0.71	[132]
			Daldiniol G (**219)**	Naphthol	n.d.	15.53 ± 0.39	[132]
	Nectriaceae	*Fusarium* sp.	Fungerin (**220**)	Imidazol type alkaloid	86.0 ± 15	1.75 ± 0.59	[64]
	Phaeosphaeriaceae	*Phaeosphaeria* sp.	Aspilactonol I (**221**)	Furanone	6.26 ± 0.15	n.d.	[133]
	Pleosporaceae	*Alternaria radicina*	Tenuazonic acid (**222**)	Pyrroline	8.13 ± 0.08	n.i.	[64]
Fungi	Pleosporaceae	*Alternaria radicina*	Epi-radicinol (**223**)	2-pyrone	6.86 ± 0.67	n.i.	[64]
			Radicinin (**224**)	2-pyrone	8.96 ± 0.97	n.i.	[64]
	Sporormiaceae	*Westerdykella nigra*	Westalsan (**225**)	Cytochalasan type alkaloid	0.088 ± 0.005	n.d.	[134]
			Phomacin B (**226**)	Cytochalasan type alkaloid	0.140 ± 0.007	n.d.	[134]
			19-hydroxy-19,20-dihydrophomacin C (**227**)	Cytochalasan type alkaloid	0.056 ± 0.003	n.d.	[134]
	Trichocomaceae	*Aspergillus* sp. 085242	Asperterpenol A (**228**)	Sestertepenoid	2.3	> 100	[135]
			Asperterpenol B (**229**)	Sestertepenoid	3.0	> 100	[135]
		*Aspergillus* sp. 16-5c	Isoaustinol (**230**)	Meroterpenoid	2.50	n.d.	[136]
			Dehydroaustin (**231**)	Meroterpenoid	0.40	n.d.	[136]
			Dehydroaustinol (**232**)	Meroterpenoid	3.00	n.d.	[136]
		*Aspergillus terreus*	Territrem D (**233**)	Meroterpenoid	4.2 ± 0.6 nM	n.d.	[66]
			Territrem E (**234**)	Meroterpenoid	4.5 ± 0.6 nM	n.d.	[66]
		*Aspergillus versicolor* Y10	Avertoxin B (**235**)	Polyketide	14.9 ^d^	n.d.	[137]
		*Penicillium* sp. SK5GW1L	Arisugacin B (**236**)	Meroterpenoid	3.03 ± 0.11	n.d.	[138]
			Territrem C (**237**)	Meroterpenoid	0.23 ± 0.09	n.d.	[138]
			Terreulactone C (**238**)	Meroterpenoid	0.028 ± 0.006	n.d.	[138]
Algae	Lessoniaceae	*Ecklonia cava*	Dieckol (**239**)	Phlorotannin	20.1 ± 0.24	2.7 ± 0.4	[139]
			8,8′-bieckol (**240**)	Phlorotannin	16.0 ± 9.4	10.9 ± 0.9	[139]
			Phlorofurofukoeckol-A (**241**)	Phlorotannin	> 50	0.9 ± 0.2	[139]
		*Eisenia bicyclis*	Phlorotannin 974-B (**242**)	Phlorotannin	1.95 ± 0.01	3.26 ± 0.08	[140]
	Sargassaceae	*Sargassum * *serratifolium*	Sargachromenol (**243**)	Terpenoid	> 50	9.4 ± 0.02	[141]
			Sargaquinoic acid (**244**)	Terpenoid	> 50	10.5 ± 0.09	[141]
		*Sargassum * *siliquastrum*	Sargachromanol I (**245**)	Terpenoid	0.79 ± 0.07	13.69 ± 5.07	[67]
			Sargachromanol G (**246**)	Terpenoid	1.81 ± 0.02	10.79 ± 0.65	[67]

**Note:** ^a^AChE from *Electrophorus electricus* unless otherwise stated. ^b^BchE from equine serum. ^c^IC_50_ values are expressed in µM, unless otherwise stated, SD values are included when available, n.d. not determined, n.i. no inhibition. ^d^*h*AChE.

**Table 15:** Potent cholinesterase inhibitors isolated from Animals reported in 2012-2022.

**Family**	**Species**	**Compound Name**	**Compound Class**	**AchE^a^** **IC_50_ ± SD^c^**	**BchE^b^** **IC_50_ ± SD^c^**	**References**
Bufonidae	*Bufo gargarizans* (toad)	Bufalin (**247**)	Bufadienolide	0.12	n.d.	[68]
Buthidae	*Buthus martensii* (scorpion)	Buthutin A (**248**)	Guanidine-type alkaloid	7.83 ± 0.06	> 30	[142]
	unidentified Caribbean coral	2-amino-3,9-dimethyl-5-methylamino-3H-1,3,4,6-tetrazacyclopent[e]azulene (**249**)	Pseudozoanthoxanthin	12.2 ±1.4^d^	14.6 ± 5.4	[143]
Axinellidae	*Axinella verrucosa* (sponge)	Stevensine (**250**)	Bromopyrrole alkaloid	7.8 ± 1.5^d^	> 100	[143]
Dictyodendrillidae	*Acanthodendrilla* sp. (sponge)	Homoaerothionin (**251**)	Bromotyrosine alkaloid	2.9 + 0.3	6.2 + 0.9	[144]
Didemnidae	*Didemnum* sp. (tunicate)	Lepadin I (**252**)	Decahydroisoquinoline alkaloid	> 100	3.1	[145]
Geodiidae	*Geodia barreti* (sponge)	6-bromoconicamin (**253**)	Indole alkaloid	> 100	14	[146]
Latrunculiidae	*Latrunculia biformis* (sponge)	Discorhabdin G (**254**)	Brominated pyrroloiminoquinone alkaloid	1.3 ± 0.2	7.0 ± 1.0	[71]
		3-dihydro-7,8-dehydrodiscorhabdin C (**255**)	Brominated pyrroloiminoquinone alkaloid	14.5 ± 1.5	15.8 ± 3.5	[71]
	*Latrunculia bocagei* (sponge)	Discorhabdin B (**256**)	Brominated pyrroloiminoquinone alkaloid	5.7 ± 0.8	>100	[71]
Plexauridae	*Eunicea knighti* (soft coral)	Asperdiol (**257**)	Diterpene cembranoid	0.358 ± 0.130	n.d.	[73]
	*Pseudoplexaura porosa* (soft coral)	14-acetoxycrassine (**258**)	Diterpene cembranoid	1.40 ± 0.113	n.d.	[73]
Pseudoceratinidae	*Pseudoceratina* cf. *purpurea* (sponge)	Purealidin Q (**259**)	Bromotyrosine alkaloid	1.2	n.d.	[72]
		Aplysamine 2 (**260**)	Bromotyrosine alkaloid	1.3	n.d.	[72]

**Note:** ^a^AChE from *Electrophorus electricus* unless otherwise stated. ^b^BChE from equine serum. ^c^IC_50_ values are expressed in µM, SD values are included when available, n.d. not determined. ^d^*h*AChE.

## LIST OF ABBREVIATIONS

ACh: Acetylcholine
AChE: Acetylcholinesterase Enzyme
AChEi: Acetylcholinesterase Inhibitor
AD: Alzheimer’s Disease
Aβ: Amyloid β Peptide
BChE: Butyrylcholinesterase Enzyme
CAS: Catalytic Active Site
EMA: European Medicines Agency
PAS: Peripheral Anionic Site
US-FDA: United States Food and Drug Administration
